# Cardiac Troponin I association with critical illness and death risk in 726 seriously ill COVID-19 patients: A retrospective cohort study

**DOI:** 10.7150/ijms.53641

**Published:** 2021-01-29

**Authors:** Huilong Chen, Xinjie Li, Tuohutaerbieke Marmar, Qiang Xu, Jing Tu, Tong Li, Jun Han, Dong Xu, Tao Shen

**Affiliations:** 1 Department and Institute of Infectious Disease, Tongji Hospital, Tongji Medical College, Huazhong University of Science and Technology, Wuhan 430030, China.; 2 Department of Microbiology and Infectious Disease Center, School of Basic Medical Sciences, Peking University, Beijing 100191, China.; 3 MHC Key Laboratory of Biosafety, National Institute for Viral Disease Control and Prevention, China CDC, Beijing 102206, China.

**Keywords:** Coronavirus disease 2019 (COVID-19), severe acute respiratory syndrome coronavirus (SARS-CoV-2), prognosis, hypersensitive cardiac troponin I (hs-cTnI), lactate dehydrogenase (LDH)

## Abstract

**Background:** For coronavirus disease 2019 (COVID-19), early identification of patients with serious symptoms at risk of critical illness and death is important for personalized treatment and balancing medical resources.

**Methods:** Demographics, clinical characteristics, and laboratory tests data from 726 patients with serious COVID-19 at Tongji Hospital (Wuhan, China) were analyzed. Patients were classified into critical group (n = 174) and severe group (n= 552), the critical group was sub-divided into survivors (n = 47) and non-survivors (n = 127).

**Results:** Multivariable analyses revealed the risk factors associated with critical illness in serious patients were: Advanced age, high respiratory rate (RR), high lactate dehydrogenase (LDH) level, high hypersensitive cardiac troponin I (hs-cTnI) level, and thrombocytopenia on admission. High hs-cTnI level was the independent risk factor of mortality among critically ill patients in the unadjusted and adjusted models. ROC curves demonstrated that hs-cTnI and LDH were predictive factors for critical illness in patients with serious COVID-19 whereas procalcitonin and D-Dimer with hs-cTnI and LDH were predictive parameters in mortality risk.

**Conclusions:** Advanced age, high RR, LDH, hs-cTnI, and thrombocytopenia, constitute risk factors for critical illness among patients with serious COVID-19, and the hs-cTnI level helps predict fatal outcomes in critically ill patients.

## Introduction

Since the first case of the novel COVID-19 pandemic was reported, the world has plunged into extreme panic. As of December 19, 2020, the total number of confirmed COVID-19 cases worldwide has exceeded 76,000,000 with more than 1,680,000 deaths 1. The virus transmission is rapid owing to population susceptibility and asymptomatic individuals who are believed to shed the virus.

Severe COVID-19 characterized by fever, cough, and fatigue may rapidly progress to irreversible adverse outcomes such as acute respiratory distress syndrome, septic shock, metabolic acidosis, blood coagulation dysfunction, and multiple organ failure 2, 3. Studies have demonstrated that patients with severe COVID-19 can experience varying degrees of damage to the lung, heart, gastrointestinal tract, kidneys, coagulation system, immune system as well as neurological impairment 4, 5. An epidemiological survey of 72314 patients reported 81%, 14%, and 5% of COVID-19 infections to be mild, severe, and critical respectively. Despite the overall case fatality rate of only 2.3%, the fatality rate in critically ill patients was as high as 49% 6, which may be higher in countries and regions with poor medical services.

The latest clinical recommendations for treatment of severe COVID-19 urge for early therapeutic interventions to lower the disproportionate risk of prolonged critical illness and death 7. However, as the ravages of COVID-19 remain unabated, medical resources are overwhelmed; therefore, optimizing the life-saving use of ventilators, renal replacement therapy equipment, and intensive care facilities is a pressing issue. Prediction of disease progression in seriously ill patients is crucial to economize medical resources and rationalize healthcare staffing.

Earlier studies, often comparing patients with mild and severe disease, indicated that advanced age, elevated CRP, D-Dimer and lymphopenia are risk factors for poor prognosis in hospitalized patients with COVID-19 3, 8. However, the key contributors to clinical course of patients already in severe conditions at admission have not been fully explored, and studies related to disease progression of critically ill patients are relatively sparse with limited cases 9, 10. Hs-cTnI has been considered as a specific and sensitive diagnostic marker for myocardial infarction, as evidence suggested that by using a hs-cTnI assay, patients with an acute myocardial infarction can be identified reliably with 100% sensitivity and 100% negative predictive value within three hours of admission 11. Early reports indicated that an elevated level of hs-cTnI could be associated with adverse outcomes of patients with COVID-19, which can act as powerful marker in managing prognosis of patients cooperating with other biomarkers, yet more evidence is required to support this suggestion 12. This study focused on predictive parameters in serious COVID-19 cases (severe and critical) to provide clinicians with a reference for substantial risk assessment for early surveillance and efficient triage, therefore maximizing on life-saving.

## Methods

### Study participants'

**Figure 1:**
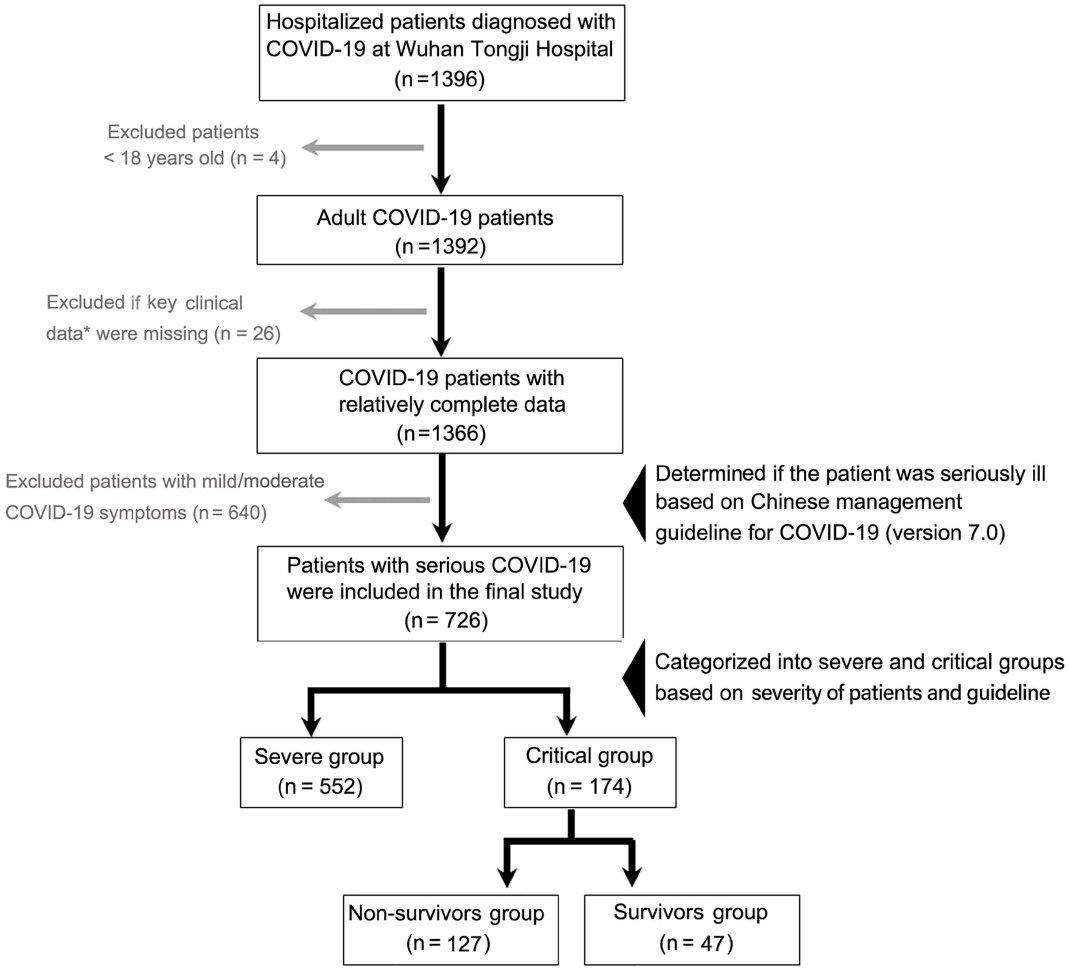
** A flow diagram for COVID-19 patient recruitment in this study.** *Key clinical data mainly refer to the time from illness onset to hospital admission, treatment options, and comorbidity details. COVID-19, Coronavirus disease 2019.

In this retrospective study, data on 1396 laboratory-confirmed COVID-19 inpatients between January 27, 2020 and February 12, 2020 was collected at Tongji Hospital, Huazhong University of Science and Technology, Wuhan, China. The patients were on follow-up for a month. The patients were diagnosed according to World Health Organization interim guidance 13, and confirmed using positive high-throughput sequencing of nasopharyngeal swab specimens or nucleic acid detection using real-time reverse transcription-polymerase chain reaction (RT-PCR). Case severity was assessed on admission based on the “Chinese management guideline for COVID-19 (version 7.0)” 14. The inclusion criteria were age ≥ 18 years, and patients with significant incomplete information and those with mild or moderate disease were excluded from this study. Finally, 726 patients with serious COVID-19 were grouped into severe cases (552) and critical cases (174), and critical cases were sub-grouped into survivors (47) and non-survivors (127). The patient recruitment process is shown in **Figure 1**.

Severe case was defined as presenting with any of the following clinical symptoms consistent with COVID-19: (1) Respiratory distress with RR ≥ 30 times/min; (2) pulse oxygen saturation (SpO_2_) ≤ 93% at rest; (3) arterial partial pressure of oxygen (PaO_2_)/fraction of inspired oxygen (FiO_2_) ≤ 300 mmHg (1 mmHg = 0.133 kPa). The critical case was defined as meeting any of the following: (1) Respiratory failure, requiring mechanical ventilation; (2) shock; (3) other organ failures, requiring intensive care unit (ICU) monitoring; or (4) death.

The study was approved by the National Health Commission of China and the Institutional Review Board of Tongji Hospital in Wuhan. Due to the urgency of the outbreak and the retrospective analysis of clinical data, the ethics committee of the designated hospital waived the informed consent requirement.

### Data collection

Data on demographics, time from illness onset to hospital admission, clinical symptoms, severity, comorbidities, treatment given, and disease outcome was collected from electronic medical records by medical professionals using standardized case report forms. Tests conducted on admission, included heart rate, blood pressure, respiratory rate, oxygen saturation, blood cell counts, blood chemistry analysis, assessment of coagulation, liver and kidney function, markers of inflammation, and markers of myocardial injury. Hs-cTnI testing was performed using the Chemiluminescent microparticle immunoassay (CMIA) on the Abbott Architect i2000. The 99^th^% URL for this method was 28 pg/mL and the lower limit of detection was 1.9 pg/mL. All data were independently recorded and verified by two technicians to ensure completeness and authenticity.

### Statistical analysis

All categorical variables were compared using the χ^2^ test or Fisher's exact test and were expressed as counts and percentages. Continuous variables were compared using the Mann-Whitney U test and were expressed as the median and inter-quartile range (IQR) as the data did not conform to a normal distribution. A single variable logistic regression analysis of 40 variables related to demographics and post-graded laboratory testing parameters was performed. Selected variables with p < 0.1 were then included in a multivariable stepwise logistic regression model with calculated OR values and 95% confidence interval (CI) to explore the risk factors associated with critical illness in hospitalized serious cases and death in critically ill patients. Four different logistic models were performed that included: Unadjusted hs-cTnI-only model 1; model 2, hs-cTnI with covariate of age; model 3, hs-cTnI with covariates of age and sex; model 4, comorbidities based on model 3. ROC curves for the variables of interest were also plotted, the area under the curve (AUC) calculated and the Youden index method used to obtain the best cutoff point. All analyses were two-sided, and p values < 0.05 were considered statistically significant. Statistical analysis was performed using SPSS version 26.0 (IBM Corp., Armonk, NY, USA).

## Results

### Demographic and clinical characteristics of seriously ill patients (severe and critical cases)

Male patients accounted for the majority (54.1%) of the 726 cases included in this study. The proportion of male patients was considerably higher in the critically ill group than in the severely ill group (62.1% vs. 51.6%, p = 0.016). The majority of patients were above 50 years old with the median age in the critically ill group being higher than in the severely ill group (IQR 68 (58, 77) vs. 65 (55, 71), p < 0.0001). Among the critically ill cases and severely ill cases, he most common symptoms included fever (85.6% vs. 86.8%), cough (78.2% vs. 78.3%) and chest distress (63.2% vs. 54.3%), while fatigue (37.9% vs. 40.2%) and diarrhea (13.8% vs. 18.3%) were relatively infrequent. Chest distress was frequently observed in critically ill patients as compared to the severely ill patients (p = 0.04), with no noticeable difference in other symptoms such as fever and cough. Hypertension was the most common comorbidity with a prevalence of 46.6% in critically ill and 41.1% in severely ill. Other comorbidities reported in included chronic obstructive lung disease (COLD) (8.6% vs. 4.3%, p = 0.029) and other diseases (including a history of surgery, hepatitis, thyroid disease, Parkinson's disease, gout, prostatic hyperplasia) which were more in the critically ill group compared to the severely ill group (24.1% vs. 17.4%, p = 0.048). Treatment administered to the two groups varied with antibiotics and antivirals being the most widely used therapies. Antibiotics had higher use in critically ill patients (94.3% vs. 76.6%) (p < 0.0001) whereas antiviral treatment had a higher usage in the severely ill (90.8% vs. 95.7%) (p = 0.015). Other therapies administered included: intravenous immunoglobulin (IVIG) (p < 0.0001), high flow nasal cannula oxygen therapy (HFNC) (p < 0.0001), invasive mechanical ventilation (IV) (p < 0.0001), non-invasive mechanical therapy (NIV) (p < 0.0001), extracorporeal membrane oxygenation (ECMO) (p = 0.003), and renal replacement therapy (RRT) (p < 0.0001) (**Table 1**).

There were statistically significant differences in various laboratory test parameters between patients in the critical group and severe group. Compared with the severe group, heart rate (p = 0.003), systolic pressure (p = 0.010), RR (p < 0.0001), WBC (p < 0.0001), neutrophil (p < 0.0001), LDH (p < 0.0001), urea (p < 0.0001), creatinine (p = 0.003), C-reactive protein (CRP) (p < 0.0001), PCT (p < 0.0001), serum ferritin (p = 0.014), hs-cTnI (p < 0.0001), IL-2R (p = 0.007), IL-6 (p < 0.0001), IL-8 (p = 0.037), IL-10 (p = 0.001), NT-proBNP (p < 0.0001) and D-Dimer (p < 0.0001) levels were raised in the critically ill group, whereas lymphocyte (p < 0.0001), platelet (p < 0.0001), albumin (p < 0.0001), and NaHCO_3_ (p = 0.006) levels were lower in the critical group. The proportion of patients with creatine kinase > 185 U/L was notably higher in the critically ill group than in the severely ill group (30.2% vs. 16.6%, p = 0.009), whereas the proportion of patients with fibrinogen > 4 g/L was much lower in the critically ill group (72.3% vs. 82.1%, p = 0.004) (**Table 1**).

### Demographic and clinical characteristics of the critically ill patients (survivors and non-survivors)

There was a similar distribution of survivors and non-survivors in terms of gender (p = 0.726) and age (p = 0.171). Patients in the survivor group appeared to have relatively more pronounced clinical symptoms as there was a higher proportion of patients with cough (89.4% vs. 74%, p = 0.038) and hypertension (59.6% vs. 41.7%, p = 0.041) than in the non-survivor group. The hs-cTnI level on admission was higher in deceased than in surviving patients (p = 0.047), which persisted even after grading the indicator (> 28 pg/mL, 42.6% vs. 23.1%, p = 0.031). In addition, the proportion of patients with creatinine > 133 μmol/L was larger in the non-survivors than in the survivors group (11.8% vs. 2.1%, p = 0.050) (**Table 2**).

### Risk factors associated with critical outcomes in seriously ill COVID-19 patients (severe and critical cases)

To explore the risk factors associated with critical outcomes in serious cases, 40 variables were included in single variable logistic regression (**Supplementary Table 1**) and were included in multivariable logistic regression where p <0. 1. Only 6 parameters that were independently statistically significant predictors of critical illness and were finally enrolled in the logistic model: Age (per year) (OR, 1.029; 95% CI, 1.011-1.048, p = 0.002), RR (> 24 times/min) (OR, 3.991; 95% CI, 2.368-6.726, p < 0.0001), lymphocyte count (< 0.8×10^9^/L) (OR, 1.519; 95% CI, 0.976 - 2.364, p = 0.064), platelet count (< 100×10^9^/L) (OR, 3.083; 95% CI, 1.412-6.731, p = 0.005), LDH (> 220 U/L) (OR, 2.848; 95% CI, 1.167-6.949, p = 0.021), hs-cTnI (>28 pg/mL) (OR, 2.899; 95% CI, 1.743 - 4.822, p < 0.0001) (**Table 3**). The evidence suggests that advanced age, RR> 24 breaths per min, lymphocyte count < 0.8×10^9^/L, platelet count < 100×10^9^/L, LDH > 220 U/L, and hs-cTnI >28 pg/mL are independent risk factors for the development of critical illness in serious patients, which were still valid in the model adjusted for sex and comorbidities (**Table 3**).

### Risk factors associated with mortality in critically ill COVID-19 patients

Single variable and multivariable logistic regression analyses performed on data of critically ill patients showed hs-cTnI level as a key independent risk factor for mortality (**Supplementary Tables 2 & 3**). Moreover, with the elevation of hs-cTnI levels, there was an upward trend in criticality and mortality (**Supplementary Figure 1**). Four hs-cTnI-based models were developed to observe the effect of hs-cTnI levels on death in critically ill patients (**Table 4**). In the unadjusted hs-cTnI logistic regression model (model 1), the risk of death in critically ill patients with a high hs-cTnI level (> 28 pg/mL) was 2.473 times higher than in patients with low levels (95% CI, 1.071-5.711, p = 0.034). In the model that included age, sex, and comorbidity effects, critical patients with high hs-cTnI level were 2.637 times more likely to die than those with low levels (95% CI,1.058-6.570, p = 0.037), which indicates that hs-cTnI levels may be useful in assessing mortality risk in critically ill patients with COVID-19. In addition, among all serious patients, therapeutic measures, such as corticosteroids (p < 0.0001), IVIG (p = 0.003), IV (p < 0.0001) and NIV (p=0.008), were needed more in patients who had high hs-cTnI level than in patients with low hs-cTnI level. Furthermore, comorbidities such as hypertension (p = 0.038) and cardiac disease (p = 0.01) were also more frequent in serious patients with high hs-cTnI level (**Figure 3**).

### Indicators predicting critical outcomes and mortality in serious patients with ROC curve

**Figure 2:**
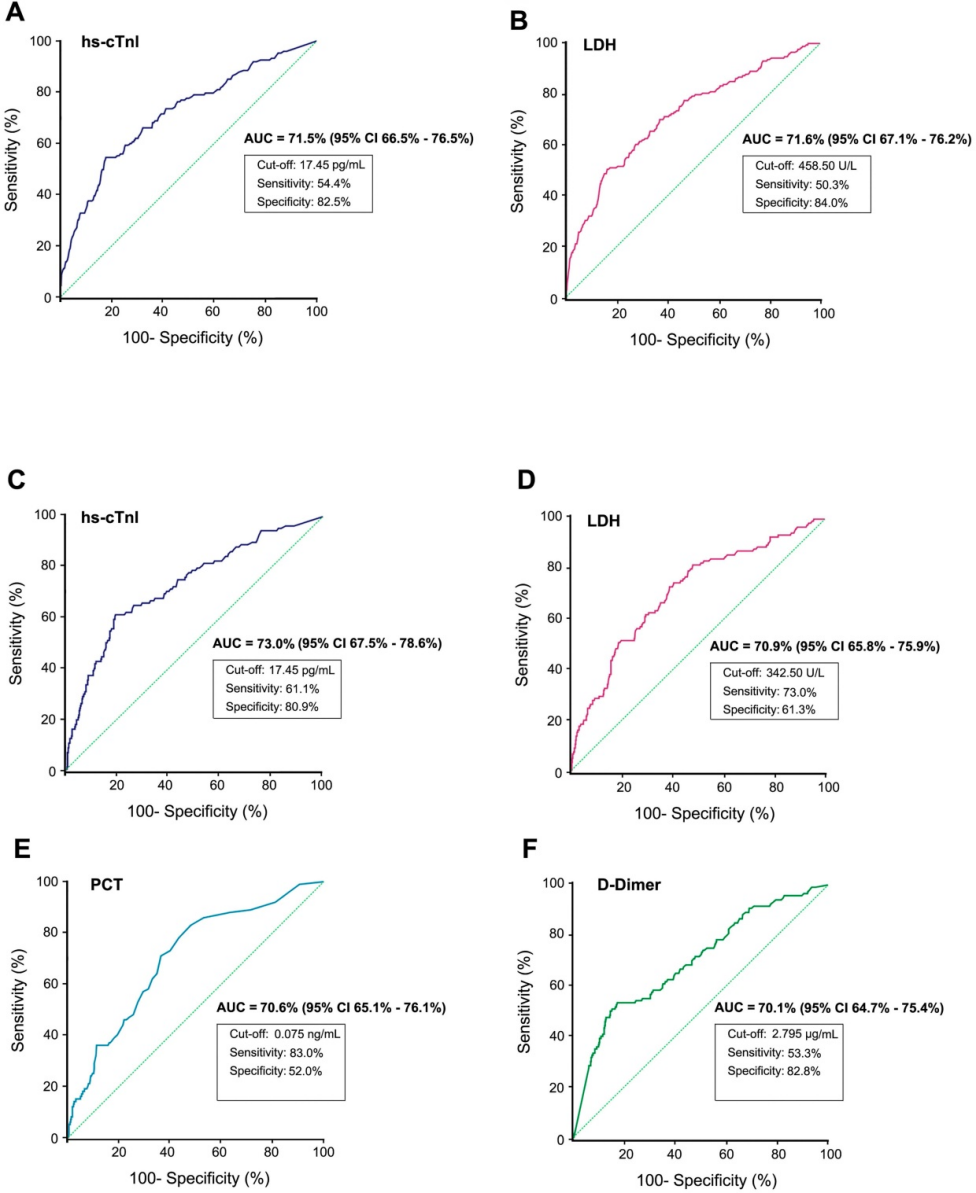
** The diagnostic value of some clinical chemistry parameters for predicting critical outcomes or fatal outcomes from serious COVID-19 were analyzed using ROC curves (only parameters with an AUC higher than 70% are listed). A-B)** The ROC curve of hs-cTnI and LDH for predicting critical illness from serious cases respectively. **C-F** The ROC curve for predicting fatal outcomes from serious COVID-19 (severe and critical cases). ROC, Receiver operating characteristic; AUC, Area under the curve; hs-cTnI, Hypersensitive cardiac troponin I; LDH, Lactate dehydrogenase; PCT, Procalcitonin.

**Figure 3:**
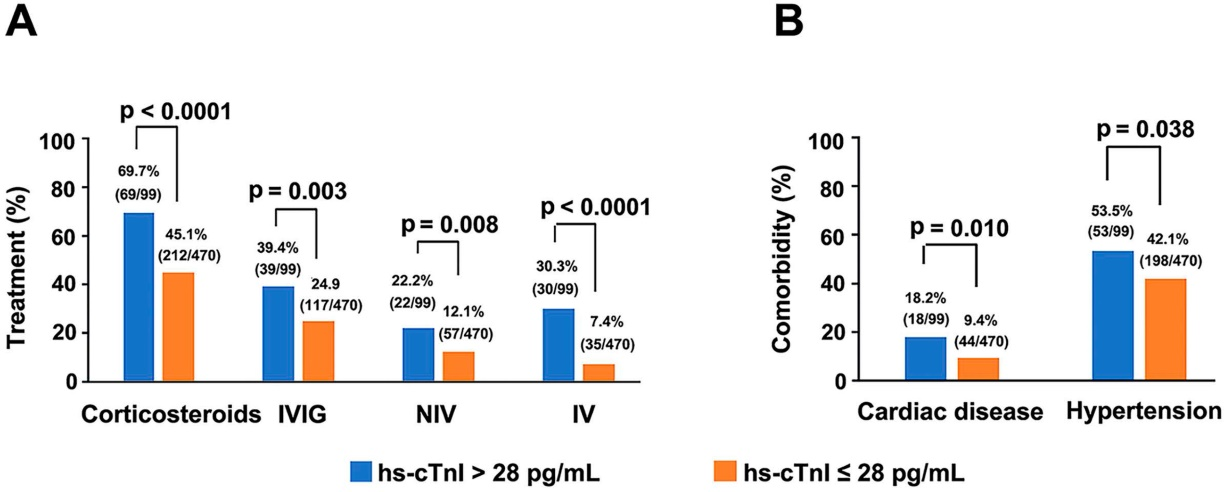
** Comparison of the main therapeutic methods (A) and comorbidities (B) between serious COVID-19 patients with initial hs-cTnI concentrations on admission > 28 pg/mL and ≤ 28 pg/mL.** Four patients suffered from cardiac diseases that were not termed as coronary heart disease. Two patients had bradycardia after cardiac pacemaker surgery (1 patient with hs-cTnI > 28 pg/mL and another with hs-cTnI ≤ 28 pg/mL), one patient had heart failure (hs-cTnI > 28 pg/mL) and one patient suffered from heart valve insufficiency (hs-cTnI > 28 pg/mL). COVID-19, Coronavirus disease 2019; IVIG, Intravenous immunoglobin; NIV, Non-invasive mechanical ventilation; IV, Invasive mechanical ventilation; hs-cTnI, Hypersensitive cardiac troponin I.

By plotting ROC curves of different indices, hs-cTnI and LDH showed positive predictive value of critical outcomes in serious COVID-19 patients. Using a cut-off of 17.45 pg/ml, the ROC curve of hs-cTnI (AUC, 71.5%; 95% CI, 66.5%-76.5%) yielded a sensitivity of 54.4% and a specificity of 82.5%, whereas the ROC curve of LDH (AUC, 71.6%; 95% CI, 67.1%-76.2%) yielded a sensitivity of 50.3% and a specificity of 84.0% at a cut-off of 458.50U/L. While predicting mortality in serious patients, in addition to hs-cTnI (AUC, 73.0%; 95% CI, 67.5%-78.6%) and LDH (AUC, 70.9%; 95% CI, 65.8%-75.9%), PCT (AUC, 70.6%; 95% CI, 65.1%-76.1%) and D-Dimer (AUC, 70.1%; 95% CI, 64.7%-75.4%) also possess promising potential (**Figure 2**). Indicators with AUC values between 60% and 70% may also hold potential value in risk prediction (**Supplementary Tables 4 & 5**). The use of different indicators can assist physicians in evaluating the risk of disease progression and death at an early stage in serious COVID-19 patients.

## Discussion

Patients diagnosed with serious COVID-19 disease require close monitoring and timely appropriate treatment to avert progression to critical stages or even death. In this study, using multivariable logistic regression analyses, advanced age, a high level of respiratory rate, LDH and hs-cTnI, and lymphopenia and thrombocytopenia at admission were found to be strongly associated with the incidence of critical ilness in patients with serious COVID-19. Also, the results revealed that hs-cTnI levels are substantially associated with mortality in critically ill patients. Moreover, it was observed that LDH and hs-cTnI were sound diagnostic markers for distinguishing between severe and critical cases. PCT and D-dimer levels in addition to LDH and hs-cTnI were also of predictive value in mortality in seriously ill COVID-19 patients.

Males accounted for a large proportion of patients admitted for severe COVID-19. The proportion of males in the critical group was significantly higher than that in the severe group, suggesting that males appear to be more susceptible to COVID-19 and are more likely to develop critical COVID-19 complications. Previous epidemiological studies also showed that the prevalence and mortality of COVID-19 was higher in males than in females 15, 16. Possible explanations are the effects of different hormones and smoking, which is more prevalent in the male population than in the female population. Smoking leads to an upregulation of angiotensin-converting enzyme 2 (ACE2) receptors, which may serve as a potential invasion points for SARS-CoV-2 17-19. The results showed that patients in severe conditions were mostly >50 years and that the critically ill group comprised more older patients than those the severe conditions group, indicating that older patients are at a higher risk of requiring critical care, which is consistent with this study's findings 20. Critically ill patients were more likely to have chest distress than severely ill patients 9. The higher incidence of COLD and other diseases in critically ill patients can be explained by the fact that patients with comorbidities are more immune-compromised. Studies have shown that a history of underlying disease acts as an influential factor in the death of critically ill COVID-19 patients 10. In this study, we did not identify a clear distinction between the severely and critically ill groups concerning carcinoma and coronary heart disease, presumably due to the relatively limited number of patients with the comorbidities. Therefore, this needs to be examined further in a larger relevant population.

Advanced age, elevated RR, LDH, hs-cTnI, decreased lymphocyte and platelet counts, constitute important risk factors for poor prognosis in serious patients. The ROC curve also demonstrated that LDH and hs-cTnI are valuable predictors of critical illness in serious cases. Critical patients are more likely than severe patients to suffer from shortness of breath and dyspnea, leading to acute respiratory distress syndrome (ARDS) and even respiratory failure 21. Therefore, intense and prompt monitoring changes in respiratory rate may satisfy the auxiliary ventilation needs in severe patients and reduce the perilous risk. Lymphocyte and platelet counts are often used to monitor viral infection in patients. Likewise, studies have shown that lymphopenia exists in the majority of patients with severe COVID-19 22, 23, and sustained low levels may further exacerbate the risk of poor prognosis and death in severe cases. LDH and hs-cTnI, as markers of myocardial injury, provide an early indicator of the extent of cardiac damage in patients 24. However, the differences between the severe and critical groups suggest that patients with serious COVID-19 may have sustained varying degrees of cardiac damage as the disease progresses. Recent studies have indicated that multiple serious sequelae persisted in patients who recovered from severe COVID-19 disease, including massive heart impairment 25, 26. Furthermore, other parameters with elevated levels such as D-dimer, cytokines (IL-2R, IL-6, IL-8, IL-10), and inflammatory factors (CRP, PCT) imply the probability of adverse prognosis in serious patients 8, 27.

Hs-cTnI also plays a pivotal role among the risk factors affecting mortality in critically ill patients. Patients with higher hs-cTnI levels are more likely to require aggressive therapeutic measures such as invasive and noninvasive mechanical ventilation and corticosteroids therapy. Hs-cTnI, has a higher sensitivity and specificity in diagnosing the myocardial injury than LDH. In most clinical situations, elevated hs-cTnI is suggestive of myocardial injury with necrosis and associated with adverse clinical outcomes 11. 10.8% of seriously ill patients in this study had a heart-related disease, and therefore elevated hs-cTnI levels in these patients are more likely to be attributed to the primary myocardial injury caused by SARS-CoV-2. A recent study has shown that SARS-CoV-2 can infect heart cells in laboratory petri-dishes, suggesting that the heart cells in COVID-19 patients may be directly infected by the virus, resulting in heart muscle damage and heart-related complications 28. Studies have reported an increased risk of death in COVID-19 patients with elevated hs-cTnI levels 12, 29^.^ Findings suggest that hs-cTnI levels had a positive predictive value in disease severity and the fatal outcomes in serious cases, even when adjusted for influences such as sex, age, and disease history. Therefore, early monitoring of cardiac injury-related markers, including hs-cTnI, can play a significant role in reducing the risk of death in serious condition.

This study has several limitations. First, there was inadequate data for some variables as this study was retrospective and the outbreak of the disease imposed a tight time frame for patient resuscitation, therefore, further validation of the specific findings is required in a prospective cohort study. Also, more specific and detailed indicator classification references should be established for COVID-19 patients. Finally, most of the data collected in this study were derived from the patient's test results on admission, however, subsequent follow-up data is required for long-term monitoring during hospitalization. At present, research on patients with severe and critical COVID-19 remains sparse and is mostly derived from single-source cases. There is a lack of appropriate pharmaceuticals and methodological support for reducing the risk of poor prognosis and death in severe as well as critically ill patients. Therefore, larger and wider case-source cohorts are required in future studies to further explore effective treatment measures for patients with serious COVID-19.

## Conclusions

This study focused on critical illness and mortality in patients with serious COVID-19. Advanced age, high RR, LDH, hs-cTnI, and low platelet counts, constitute important risk factors for critical illness among serious cases of COVID-19 on admission, and the hs-cTnI level helps predict fatal outcomes in critically ill patients. It aims to provide clinicians with a practical reference for risk assessment among serious patients on admission, early graded care, and rational allocation of medical resources for seriously ill COVID 19 patients.

## Supplementary Material

Supplementary figures and tables.

## Figures and Tables

**Table 1:** Demographic and clinical characteristics of patients diagnosed with severe and critical COVID-19 on admission

Variables	Critical patients (n = 174)	Severe patients (n = 552)	*p* value
**Sex, n (%)**			
Male	108 (62.1)	285 (51.6)	**0.016**
**Age (yrs), median (IQR) or n (%)**	68 (58, 77)	65 (55, 71)	**< 0.0001**
> 50	162 (93.1)	454 (82.2)	**< 0.0001**
**Time from illness onset to hospital admission (days), median (IQR)**	10 (7, 15)	10 (7, 14)	0.958
**Symptom, n (%)**			
Fever (temperature ≥ 37.3 °C)	149 (85.6)	479 (86.8)	0.700
Diarrhea	24 (13.8)	101 (18.3)	0.170
Cough	136 (78.2)	432 (78.3)	0.729
Fatigue	66 (37.9)	222 (40.2)	0.591
Chest distress	110 (63.2)	300 (54.3)	**0.040**
**Treatment, n (%)**			
Antibiotics	164 (94.3)	423 (76.6)	**< 0.0001**
Antiviral treatment	158 (90.8)	528 (95.7)	**0.015**
Corticosteroids	139 (79.9)	240 (43.5)	**< 0.0001**
IVIG	86 (49.4)	123 (22.3)	**< 0.0001**
HFNC	29 (16.7)	26 (4.7)	**< 0.0001**
NIV	75 (43.1)	24 (4.3)	**< 0.0001**
IV	75 (43.1)	2 (0.4)	**< 0.0001**
ECMO	4 (2.3)	1 (0.2)	**0.003**
RRT	9 (6.0)	1 (0.3)	**< 0.0001**
**Comorbidity, n (%)**			
Hypertension	81 (46.6)	227 (41.1)	0.206
Diabetes	40 (23.0)	100 (18.1)	0.155
COLD	15 (8.6)	24 (4.3)	**0.029**
CHD	16 (9.2)	55 (10.0)	0.766
CKD	5 (2.9)	8 (1.4)	0.217
Carcinoma	7 (4.0)	19 (3.4)	0.719
Other disease	42 (24.1)	96 (17.4)	**0.048**
**Physiological parameter, median (IQR) or n/N (%)**			
Heart rate, per min	95 (81, 108)	88 (80, 102)	**0.003**
< 60	0/173 (0)	10/552 (1.8)	**0.001**
60-100	104/173 (60.1)	395/552 (71.6)	
> 100	69/173 (39.9)	147/552 (26.6)	
Systolic pressure	132 (120, 150)	129 (119, 141)	**0.010**
> 120	128/172 (74.4)	381/552 (69.0)	0.176
Diastolic pressure	80 (72, 90)	79 (70, 88)	0.107
≤ 80	88/172 (51.2)	309/552 (56.0)	0.268
RR, breaths per min	22 (20, 26)	20 (20, 22)	**< 0.0001**
> 24	56/171 (32.7)	58/552 (10.5)	**< 0.0001**
SPO_2_	92 (83, 97)	95 (92, 97)	**< 0.0001**
≤ 95	88/138 (63.8)	277/523 (53.0)	**0.023**
**Biological parameters, median (IQR) or n/N (%)**			
WBC, ×10^9^/L	7.3 (5.4, 10.9)	5.7 (4.5, 7.5)	**< 0.0001**
< 4	17/174 (9.8)	98 /551 (17.8)	**< 0.0001**
4-10	106/174 (60.9)	392/551 (71.1)	
> 10	51/174 (29.3)	61/551 (11.1)	
Neutrophil, ×10^9^/L	6.1 (4.0, 9.6)	4.0 (2.9, 5.7)	**< 0.0001**
< 1.8	4/174 (2.3)	36/551 (6.5)	**< 0.0001**
1.8-6.3	86/174 (49.4)	402/551 (73.0)	
> 6.3	84/174 (48.3)	113/551 (20.5)	
Lymphocyte, ×10^9^/L	0.70(0.50, 0.95)	0.95 (0.66, 1.35)	**< 0.0001**
≤ 0.8	113/174 (64.9)	223/551 (40.5)	**< 0.0001**
Hemoglobin, g/L	129 (117, 141)	128 (117, 139)	0.378
< 110	23/174 (13.2)	72/551 (13.1)	0.157
110-150	132/174 (75.9)	443/551 (80.4)	
> 150	19/174 (10.9)	36/551 (6.5)	
Platelet, ×10^9^/L	172.5 (121.7, 242.7)	219.0 (160.0, 294.0)	**< 0.0001**
≤100	27/174 (15.5)	21/550 (3.8)	**< 0.0001**
Albumin, g/L	31.3 (28.8, 34.5)	33.5 (30.6, 36.6)	**< 0.0001**
< 35	136/173 (78.6)	339/551 (61.5)	**< 0.0001**
35-55	36/173 (20.8)	212/551 (38.5)	
> 55	1/173 (0.6)	0/551 (0)	
LDH, U/L	459.0 (314.0, 588.5)	302.0 (247.0, 413.0)	**< 0.0001**
> 220	163/173 (94.2)	464/550 (84.5)	**0.001**
NaHCO_3_, mmol/L	22.9 (20.7, 25.2)	23.8 (21.9, 25.1)	**0.006**
< 22	69/174 (39.7)	142/551 (25.8)	**0.001**
22-27	86/174 (49.4)	360/551 (65.3)	
> 27	19/174 (10.9)	49/551 (8.9)	
Urea, mmol/L	5.8 (4.6, 8.3)	4.6 (3.6, 6.3)	**< 0.0001**
> 7.5	58/174 (33.3)	82/551 (14.9)	**< 0.0001**
Creatinine, μmol/L	78.0 (60.0, 97.3)	71.0 (58.0, 87.0)	**0.003**
> 133	16/174 (9.2)	26/551 (4.7)	**0.028**
Creatine kinase, U/L	99.5 (46.8, 236.3)	66.0 (43.0, 139.0)	0.053
> 185	26/86 (30.2)	33/199 (16.6)	**0.009**
CRP, mg/L	87.3 (39.3, 142.7)	43.0 (11.0, 91.6)	**< 0.0001**
< 10	14/166 (8.4)	123/535 (23.0)	**< 0.0001**
10-50	37/166 (22.3)	167/535 (31.2)	
> 50	115/166 (69.3)	245/535 (45.8)	
PCT, ng/mL	0.16 (0.08, 0.37)	0.07 (0.04, 0.15)	**< 0.0001**
< 0.5	115/138 (83.3)	378/406 (93.1)	**0.001**
0.5-2	15/138 (10.9)	22/406 (5.4)	
> 2	8/138 (5.8)	6/406 (1.5)	
SF, μg/L	1130.9 (606.0, 1645.7)	801.6 (480.1, 1415.0)	**0.014**
> 300	73/79 (92.4)	166/192 (86.5)	0.168
Fibrinogen, g/L	5.1 (3.9, 6.5)	5.4 (4.4, 6.3)	0.168
< 2	8/148 (5.4)	6/436 (1.4)	**0.004**
2-4	33/148 (22.3)	72/436 (16.5)	
> 4	107/148 (72.3)	358/436 (82.1)	
Hs-cTnI, pg/mL	18.8 (7.1, 54.9)	6.2 (3.1, 13.8)	**< 0.0001**
> 28	55/147 (37.4)	44/422 (10.4)	**< 0.0001**
IL-2R, U/mL	1051.5 (617.0, 1447.7)	806.0 (609.5, 1106.0)	**0.007**
< 220	1/84(1.2)	6/228 (2.6)	0.259
220-710	24/84 (28.6)	84/228 (36.8)	
> 710	59/84 (70.2)	138/228 (60.5)	
IL-6, pg/mL	43.4 (18.3, 82.3)	22.8 (7.3, 53.6)	**< 0.0001**
> 7	71/83 (85.5)	75/232 (75.4)	0.056
IL-8, pg/mL	21.7 (12.8, 32.7)	17.3 (10.1, 28.1)	**0.037**
> 62	10/84 (11.9)	16/228 (7.0)	0.166
IL-10, pg/mL	7.7 (5.3, 12.0)	5.5 (5.0, 9.2)	**0.001**
> 9	33/83 (39.8)	59/228 (25.9)	**0.018**
NT-proBNP, pg/mL	455.5 (168.2, 1269.0)	178.5 (68.7, 449.5)	**< 0.0001**
< 500	69/136 (50.7)	292/370 (78.9)	**< 0.0001**
500-1000	29/136 (21.3)	45/370 (12.2)	
> 1000	38/136 (27.9)	33/370 (8.9)	
D-Dimer, μg/mL	2.5 (0.91, 21.0)	1.0 (5.2, 2.1)	**< 0.0001**
< 0.5	17/162 (10.5)	118/504 (23.4)	**< 0.0001**
0.5-1	28/162 (17.3)	136/504 (27.0)	
> 1	117/162 (72.2)	250/504 (49.6)	

Data is shown as the median (IQR), n (%), or n/N (%), where N is the total number with the available data. IQR, Interquartile range; IVIG, Intravenous immunoglobin; HFNC, High-flow nasal cannula oxygen therapy; NIV, Non-invasive mechanical ventilation; IV, Invasive mechanical ventilation; RRT, Renal replacement therapy; COLD, Chronic obstructive lung disease; CHD, Coronary heart disease; CKD, Chronic kidney disease; ECMO, Extracorporeal membrane oxygenation; RR, Respiratory rate; SpO_2_, Pulse oxygen saturation; SF, Serum ferritin; Hs-cTnI, Hypersensitive cardiac troponin I; WBC, White blood cell count; NT-proBNP, N terminal pro B type natriuretic peptide; PCT, Procalcitonin; CRP, C-reactive protein; LDH, Lactate dehydrogenase.

**Table 2:** Demographic and clinical characteristics of survivors and non-survivors among critical COVID-19 patients on admission

Variables	Non-survivors (n = 127)	Survivors (n = 47)	*p* value
**Sex, n (%)**			
Male	80 (63.0)	28 (59.6)	0.726
**Age (yrs), median (IQR) or n (%)**	68 (59, 77)	66 (57, 74)	0.171
> 50	119 (93.7)	43 (91.5)	0.609
**Time from illness onset to hospital admission (days), median (IQR)**	10 (7, 15)	12 (9, 14)	0.477
**Symptom, n (%)**			
Fever (temperature ≥ 37.3 °C)	107 (84.3)	42 (89.4)	0.473
Diarrhea	18 (14.2)	6 (12.8)	1.000
Cough	94 (74)	42 (89.4)	**0.038**
Fatigue	47 (37)	19 (40.4)	0.726
Chest distress	79 (62.2)	31 (66)	0.725
**Treatment, n (%)**			
Antibiotics	120 (94.5)	44 (93.6)	1.000
Antiviral treatment	113 (89)	45 (95.7)	0.241
Corticosteroids	104 (81.9)	35 (74.5)	0.292
IVIG	61 (48)	25 (53.2)	0.610
HFNC	17 (13.4)	12 (22.5)	0.068
NIV	58 (45.7)	17 (36.2)	0.303
IV	58 (45.7)	17 (36.2)	0.303
ECMO	3 (2.4)	1 (2.1)	1.000
RRT	5 (4.7)	4 (9.1)	0.449
**Comorbidity, n (%)**			
Hypertension	53 (41.7)	28 (59.6)	**0.041**
Diabetes	29 (22.8)	11 (23.4)	1.000
COLD	12 (9.4)	3 (6.4)	0.762
CHD	12 (9.4)	4 (8.5)	1.000
CKD	5 (3.9)	0 (0)	0.325
Carcinoma	6 (4.7)	1 (2.1)	0.676
Other disease	33 (26)	9 (19.1)	0.427
**Physiological parameter, median (IQR) or n/N (%)**			
Heart rate, per min	95 (82, 108)	95 (81, 108)	0.879
60-100	73/126 (57.9)	31/47 (66.0)	0.338
> 100	53/126 (42.1)	16/47 (34.0)	
Systolic pressure	132 (120, 146)	134 (120, 151)	0.617
> 120	94/126 (74.6)	34/46 (73.9)	0.927
Diastolic pressure	79 (72, 90)	83 (75, 92)	0.246
≤ 80	69/126 (54.8)	19/46 (41.3)	0.118
RR, breaths per min	21 (20, 27)	22 (20, 26)	0.857
> 24	41/125 (32.8)	15/46 (32.6)	0.981
SPO2	90.5 (81.8, 97.0)	94.5 (86.3, 97.0)	0.171
≤ 95	64/98 (65.3)	24/40 (60.0)	0.556
**Biological parameters, median (IQR) or n/N (%)**			
WBC, ×10^9^/L	7.7 (5.7, 11.3)	7.3 (4.6, 9.5)	0.241
< 4	10/127 (7.9)	7/47 (14.9)	0.198
4-10	76/127 (59.8)	30/47 (63.8)	
> 10	41/127 (32.3)	10/47 (21.3)	
Neutrophil, ×10^9^/L	6.4 (4.2, 10.0)	6.0 (3.5, 8.0)	0.233
< 1.8	1/127 (0.8)	3/47 (6.4)	0.076
1.8-6.3	62/127 (48.8)	24/47 (51.1)	
> 6.3	64/127 (50.4)	20/47 (42.6)	
Lymphocyte, ×10^9^/L	0.67 (0.47, 0.90)	0.73 (0.53, 1.00)	0.109
≤ 0.8	87/127 (68.5)	26/47 (55.3)	0.106
Hemoglobin, g/L	129 (117, 141)	128 (117, 141)	0.634
< 110	18/127 (14.2)	5/47 (10.6)	0.819
110-150	95/127 (74.8)	37/47 (78.7)	
> 150	14/127 (11.0)	5/47 (10.6)	
Platelet, ×10^9^/L	167 (120, 248)	174 (122, 240)	0.708
≤ 100	20/127 (15.7)	7/47 (14.9)	0.890
Albumin, g/L	31.3 (28.3, 34.6)	31.5 (30.0, 34.4)	0.186
< 35	100/126 (79.4)	36/47 (76.6)	0.257
35-55	26/126 (20.6)	10/47 (21.3)	
> 55	0/126 (0)	1/47 (2.1)	
LDH, U/L	459.0 (323.2, 574.0)	445.0 (281.0, 609.0)	0.816
> 220	118/126 (93.7)	45/47 (95.7)	0.600
NaHCO_3_, mmol/L	22.6 (20.5, 25.0)	23.2 (20.7, 25.6)	0.394
< 22	52/127 (40.9)	17/47 (36.2)	0.805
22-27	62/127 (48.8)	24/47 (51.1)	
> 27	13/127 (10.2)	6/47 (12.8)	
Urea, mmol/L	5.9 (4.7, 9.0)	5.6 (3.9, 7.7)	0.178
> 7.5	46/127 (36.2)	12/47 (25.5)	0.184
Creatinine, μmol/L	77.0 (60.0, 97.0)	79.0 (58.0, 101.0)	0.926
> 133	15/127 (11.8)	1/47 (2.1)	**0.050**
Creatine kinase, U/L	106.0 (48.0, 246.0)	73.0 (44.5, 231.0)	0.775
> 185	18/61 (29.5)	8/25 (32.0)	0.819
CRP, mg/L	92.4 (39.8, 152.8)	66.4 (33.1, 117.2)	0.089
< 10	10/120 (8.3)	4/45 (8.9)	0.903
10-50	26/120 (21.5)	11/45 (24.4)	
> 50	85120 (70.2)	30 /45 (66.7)	
PCT, ng/mL	0.16 (0.09, 0.41)	0.16 (0.07, 0.29)	0.162
< 0.5	81/100 (81.0)	34/38 (89.5)	0.194
0.5 - 2	11/100 (11.0)	4 /38 (10.5)	
> 2	8/100 (8.0)	0 /38 (0)	
SF, μg/L	1162.5 (637.8, 1728.7)	893.6 (549.3, 142.0)	0.274
> 300	54/57 (94.7)	19/22 (86.4)	0.208
Fibrinogen, g/L	5.4 (4.0, 6.7)	5.1 (3.7, 5.9)	0.554
< 2	7/103 (6.8)	1/45 (2.2)	0.276
2-4	20/103 (19.4)	13/45 (28.9)	
> 4	76/103 (73.8)	31/45 (68.9)	
Hs-cTnI, pg/mL	22.5 (7.6, 64.0)	11.8 (4.2, 26.1)	**0.047**
> 28	46/108 (42.6)	9 /39 (23.1)	**0.031**
IL-2R, U/mL	1094.5 (642.0, 1534.0)	1009.5 (608.2, 1165.5)	0.289
< 220	1/60 (1.7)	0/24 (0)	0.444
220 - 710	15/60 (25)	9/24 (37.5)	
> 710	44/60 (73.3)	15/24 (62.5)	
IL-6, pg/mL	51.4 (18.3, 119.6)	35.6 (15.2, 48.1)	0.062
> 7	52/59 (88.1)	19/24 (79.2)	0.292
IL-8, pg/mL	22.9 (13.4, 38.9)	17.1 (10.4, 28.0)	0.148
> 62	8/60 (13.3)	2/24 (8.3)	0.523
IL-10, pg/mL	8.3 (5.4, 10.8)	6.7 (5.0, 15.8)	0.868
> 9	23/59 (39.0)	10/24 (41.7)	0.821
NT-proBNP, pg/mL	565.0 (186.7, 1338.5)	311.0 (121.0, 799.5)	0.092
< 500	48/104 (46.2)	21/32 (65.6)	0.142
500-1000	25/104 (24.0)	4/32 (12.5)	
> 1000	31/104 (29.8)	7/32 (21.9)	
D-Dimer, μg/mL	3.1 (0.94, 21.0)	1.7 (0.66, 21.0)	0.251
< 0.5	10/120 (8.3)	7/42 (16.7)	0.314
0.5-1	21/120 (17.5)	7/42 (16.7)	
> 1	89/120 (74.2)	28/42 (66.7)	

Data is shown as the median (IQR), n (%), or n/N (%), where N is the total number with the available data. IQR, Interquartile range; IVIG, Intravenous immunoglobin; HFNC, High-flow nasal cannula oxygen therapy; NIV, Non-invasive mechanical ventilation; IV, Invasive mechanical ventilation; RRT, Renal replacement therapy; COLD, Chronic obstructive lung disease; CHD, Coronary heart disease; CKD, Chronic kidney disease; ECMO, Extracorporeal membrane oxygenation; RR, Respiratory rate; SpO_2_, Pulse oxygen saturation; SF, Serum ferritin; Hs-cTnI, Hypersensitive cardiac troponin I; WBC, White blood cell count; NT-proBNP, N terminal pro B type natriuretic peptide; PCT, Procalcitonin; CRP, C-reactive protein; LDH, Lactate dehydrogenase.

**Table 3:** Multivariable logistic regression model for predicting the development of critical cases among 726 serious COVID-19 patients

Variables	Crude Odds ratio	95% CI	*p* value	Adjusted Odds ratio^a^	95% CI	*p* value
Age, year	1.029	1.011-1.048	0.002	1.029	1.010-1.048	**0.003**
RR, breaths per min (> 24 vs ≤ 24)	3.991	2.368-6.726	<0.0001	3.830	2.246-6.530	**<0.0001**
Lymphocyte count, × 10^9^/L (< 0.8 vs ≥ 0.8)	1.519	0.976-2.364	0.064	1.452	0.926-2.276	0.104
Platelet count, × 10^9^/L (< 100 vs ≥ 100)	3.083	1.412-6.731	0.005	3.036	1.355-6.799	**0.007**
LDH, U/L (> 220 vs ≤ 220)	2.848	1.167-6.949	0.021	3.081	1.246-7.623	**0.015**
Hs-cTnI, pg/mL (> 28 vs ≤ 28)	2.899	1.743-4.822	<0.0001	3.029	1.794-5.112	**<0.0001**
Constant	0.009	-	<0.0001	0.019	-	**0.002**

RR, Respiratory rate; LDH, Lactate dehydrogenase; Hs-cTnI, Hypersensitive cardiac troponin I; CI, Confidence interval. ^a^Adjusted for sex and comorbidities, including hypertension, diabetes, chronic obstructive lung disease, coronary heart disease, chronic kidney disease, carcinoma and other disease.

**Table 4:** Logistic regression model evaluating hs-cTnI levels for death risk among critical COVID-19 patients

Model	Odds ratio (> 28 vs ≤ 28 pg/mL)	95% CI	*p* value
Model 1	2.473	1.071-5.711	**0.034**
Model 2	2.210	0.944-5.177	0.068
Model 3	2.284	0.969-5.384	0.059
Model 4	2.637	1.058-6.570	**0.037**

Model 1 is the unadjusted hypersensitive troponin I-only model. Model 2 includes hypersensitive troponin I with the additional covariate of age. Model 3 includes hypersensitive troponin I with the additional covariates of age and sex. Model 4 includes hypertension, diabetes, chronic obstructive lung disease, coronary heart disease, chronic kidney disease, carcinoma and other disease based on model 3. hs-cTnI, Hypersensitive cardiac troponin I; CI, Confidence interval.

## Abbreviations

COVID-19: coronavirus disease 2019
SARS-CoV-2: severe acute respiratory syndrome coronavirus
RT-PCR: reverse transcription-polymerase chain reaction
ICU: intensive care unit
PaO2: partial pressure of oxygen
FiO2: fraction of inspiration oxygen
ECMO: extracorporeal membrane oxygenation
CMIA: chemiluminescent microparticle immunoassay
IQR: interquartile range
CI: confidence interval
ROC: receiver operating characteristic
AUC: area under curve
OR: odds ratio
ACE2: angiotensin-converting enzyme 2
COLD: chronic obstructive lung disease
HFNC: high-flow nasal cannula oxygen therapy
IVIG: Intravenous immunoglobin
IV: Invasive mechanical ventilation
NIV: non-invasive mechanical ventilation
RRT: renal replacement therapy
WBC: white blood cell count
Hs-cTnI: hypersensitive cardiac troponin I
LDH: Lactate dehydrogenase
CPR: C-reactive protein
PCT: procalcitonin
ARDS: acute respiratory distress syndrome
