# Supplementary material for: Cardiac Troponin I association with critical illness and death risk in 726 seriously ill COVID-19 patients: A retrospective cohort study
Source: Int J Med Sci. 2021 Jan 29;18(6):1474–83. doi: 10.7150/ijms.53641 (PMC7893554; doi:10.7150/ijms.53641)
Supplement: Supplementary file 1 — Supplementary figures and tables. [file ijmsv18p1474s1.pdf]

Supplementary data

Supplementary Table 1. Single variable logistic regression model for predicting the risk of critical outcomes among seriously ill COVID-19 patients

Variables	Odds ratio	95% CI	p value
Sex (male vs female)	1.533	1.082 - 2.173	***0.016***
Age, per year	1.034	1.018 - 1.049	***<0.0001***
Fever (yes vs no)	0.908	0.556 - 1.483	*0.700*
Diarrhea (yes vs no)	0.714	0.441 - 1.157	*0.171*
Cough (yes vs no)	0.974	0.648 - 1.463	*0.899*
Fatigue (yes vs no)	0.908	0.640 - 1.289	*0.591*
Chest distress (yes vs no)	1.444	1.017 - 2.050	***0.040***
SPO_2 (< 95 vs ≥ 95)	1.563	1.061 - 2.302	***0.024***
Systolic pressure (> 120 vs ≤ 120)	1.306	0.887 - 1.923	*0.177*
Diastolic pressure (> 80 vs ≤ 80)	1.214	0.861 - 1.710	*0.268*
RR, breaths per min (> 24 vs ≤ 24)	4.148	2.726 - 6.309	***<0.0001***
Hypertension (yes vs no)	1.247	0.885 - 1.757	*0.207*
COLD (yes vs no)	2.075	1.063 - 4.052	***0.032***
CHD (yes vs no)	0.915	0.510 - 1.642	*0.766*
Diabetes (yes vs no)	1.349	0.892 - 2.042	*0.156*
CKD (yes vs no)	2.012	0.649 - 6.232	*0.226*
Carcinoma (yes vs no)	1.176	0.486 - 2.846	*0.719*
Other disease (yes vs no)	1.511	1.002 - 2.280	***0.049***
WBC count, × 10^9/L			
4 - 10 vs ≤ 4	1.559	0.892 - 2.723	*0.119*
> 10 vs ≤ 4	4.820	2.544 - 9.097	***<0.0001***
Neutrophil count, × 10^9/L			
1.8 - 6.3 vs < 1.8	1.925	0.668 - 5.551	*0.225*
> 6.3 vs < 1.8	6.690	2.293 - 19.522	***0.001***
Lymphocyte count, × 10^9/L (≤ 0.8 vs > 0.8)	2.725	1.911 - 3.885	***<0.0001***
Hemoglobin, g/L			
110 - 150 vs < 110	0.933	0.561 - 1.550	*0.788*
> 150 vs < 110	1.652	0.798 - 3.420	*0.176*
Platelet count, × 10^9/L (≤ 100 vs > 100)	4.627	2.542 - 8.421	***<0.0001***
Albumin, g/L (≥ 35 vs < 35)	0.423	0.282 - 0.635	***<0.0001***
Creatine kinase, U/L (> 185 vs ≤ 185)	2.180	1.205 - 3.943	***0.010***
LDH, U/L (> 220 vs ≤ 220)	2.980	1.511 - 5.876	***0.002***
$NaHCO_3$, mmol/L (22-27 vs < 22)	0.492	0.339 - 0.713	***<0.0001***
Urea, mmol/L (> 7.5 vs ≤ 7.5)	2.860	1.931 - 4.236	***<0.0001***
Creatinine, μmol/L (> 133 vs ≤ 133)	2.045	1.070 - 3.908	***0.030***
CRP, mg/L			
10 - 50 vs < 10	1.947	1.008 - 3.757	***0.047***
> 50 vs < 10	4.124	2.273 - 7.481	***<0.0001***

PCT, ng/mL			
0.5 - 2 vs < 0.5	2.241	1.126 - 4.462	***0.022***
> 2 vs < 0.5	4.383	1.490 - 12.891	***0.007***
SF, μg/L (> 300 vs ≤ 300)	1.906	0.752 - 4.827	*0.174*
Hs-cTnI, pg/mL (> 28 vs ≤ 28)	4.988	3.155 - 7.887	***<0.0001***
NT-proBNP, pg/mL			
500 - 1000 vs < 500	2.727	1.597 - 4.648	***<0.0001***
> 1000 vs < 500	4.873	2.854 - 8.321	***<0.0001***
Fibrinogen, g/L			
2 - 4 vs < 2	0.344	0.110 - 1.070	*0.065*
> 4 vs < 2	0.224	0.076 - 0.660	***0.007***
D-Dimer, μg/mL			
0.5 - 1 vs < 0.5	1.429	0.745 - 2.741	*0.283*
> 1 vs < 0.5	3.248	1.867 - 5.652	***<0.0001***
IL-2R, U/mL			
220-710 vs < 220	1.714	0.197 - 14.940	*0.626*
>710 vs < 220	2.565	0.302 - 21.778	*0.388*
IL-6, pg/mL (> 7 vs ≤ 7)	1.927	0.975 - 3.807	*0.059*
IL-8, pg/mL (> 62 vs ≤ 62)	1.791	0.778 - 4.120	*0.171*
IL-10, pg/mL (> 9 vs ≤ 9)	1.891	1.113 - 3.212	***0.019***

SpO_2, Pulse oxygen saturation; RR, Respiratory rate; COLD, Chronic obstructive lung disease; CHD, Coronary heart disease; CKD, Chronic kidney disease; WBC, White blood cell; SF, Serum ferritin; Hs-cTnI, Hypersensitive cardiac troponin I; NT-proBNP, N terminal pro B type natriuretic peptide; PCT, Procalcitonin; CRP, C-reactive protein; LDH, Lactate dehydrogenase; CI, Confidence interval.

Supplementary Table 2. Single variable logistic regression model for predicting the risk of fatal outcomes among critically ill COVID-19 patients (only variables with $p<0.1$ were listed)

Variables	Odds ratio	95% CI	p value
Cough (yes vs no)	0.339	0.124 - 0.930	***0.036***
Hypertension (yes vs no)	0.486	0.246 - 0.960	***0.038***
WBC count, × 10^9/L			
4 - 10 vs ≤ 4	1.773	0.618 - 5.089	*0.287*
> 10 vs ≤ 4	2.870	0.875 - 9.413	*0.082*
Neutrophil cell count, × 10^9/L			
1.8 - 6.3 vs < 1.8	7.750	0.768 - 78.210	*0.083*
> 6.3 vs < 1.8	9.600	0.945 - 97.511	*0.056*
Creatinine, μmol/L (> 133 vs ≤ 133)	6.161	0.791 - 48.006	*0.083*
Hs-cTnI, pg/mL (> 28 vs ≤ 28)	2.473	1.071 - 5.711	***0.034***
NT-proBNP, pg/mL			
500 - 1000 vs < 500	2.734	0.846 - 8.841	*0.093*
> 1000 vs < 500	1.937	0.736 - 5.097	*0.180*

WBC, White blood cell; Hs-cTnI, Hypersensitive cardiac troponin I; NT-proBNP, N terminal pro B type natriuretic peptide; CI, Confidence interval.

Supplementary Table 3. Multivariable logistic regression model for predicting the risk of fatal outcomes among critically ill COVID-19 patients

**Variables**	**Odds ratio**	**95% CI**	**p value**
Age, year	1.034	1.000 - 1.069	***0.002***
Hs-cTnI, pg/mL (> 28 vs ≤ 28)	2.210	0.944 - 5.177	***<0.068***
Constant	0.231		*0.192*

Hs-cTnI, Hypersensitive cardiac troponin I. CI, Confidence interval; OR, Odds ratio.

Supplementary Table 4. ROC curve information for possible indicators for predicting critical outcomes among seriously ill COVID-19 patients with AUC values between 60% and 70%

**Variables**	**AUC**	**95% CI**	**Cut-off**	**Sensitivity (%)**	**Specificity (%)**
Age (year)	0.603	0.554 - 0.651	69.5	44.8	71.2
SPO_2 (%)	0.623	0.562 - 0.683	88.5	40.6	89.1
RR (t/m)	0.650	0.600 - 0.701	25.5	29.8	93.8
WBC (10^9/L)	0.659	0.610 - 0.707	6.945	56.9	70.1
Neutrophil (10^9/L)	0.685	0.638 - 0.732	5.585	56.3	73.7
Lymphocyte (10^9/L)	0.649	0.602 - 0.696	0.725	56.9	67.7
Platelet (10^9/L)	0.639	0.591 - 0.688	177.50	55.2	68.5
Albumin (g/L)	0.631	0.584 - 0.679	31.95	56.6	64.6
Urea (mmol/L)	0.644	0.596 - 0.693	4.95	69.5	56.8
CRP (mg/L)	0.666	0.619 - 0.712	64.45	62.0	63.6
PCT (ng/ mL)	0.699	0.649 - 0.749	0.075	80.4	54.4
NT-proBNP (pg/mL)	0.679	0.626 - 0.733	501.0	49.3	78.9
D-Dimer (ug/mL)	0.693	0.644 - 0.742	3.18	46.3	87.3
IL-2R (U/mL)	0.600	0.526 - 0.675	985.0	57.1	67.1
IL-6 (pg/mL)	0.639	0.569 - 0.709	32.615	66.3	60.3
IL-8 (pg/mL)	0.605	0.528 - 0.682	20.55	61.7	61.8
IL-10 (pg/mL)	0.629	0.561 - 0.696	5.05	79.5	46.3

SpO_2, Pulse oxygen saturation; RR, Respiratory rate; WBC, White blood cell; NT-proBNP, N terminal pro B type natriuretic peptide; PCT, Procalcitonin; CRP, C-reactive protein; CI, Confidence interval.

Supplementary Table 5. ROC curve information for possible indicators for predicting fatal outcomes among seriously ill COVID-19 patients with AUC values between 60% and 70%

Variable	AUC	95% CI	Cut-off	Sensitivity (%)	Specificity (%)
Age (year)	0.615	0.561 - 0.670	69.5	47.2	70.2
SPO_2 (%)	0.640	0.569 - 0.711	90.5	50.0	82.6
RR (t/m)	0.641	0.584 - 0.698	23.5	27.6	84.3
WBC (10^9/L)	0.667	0.614 - 0.721	7.65	51.2	75.6
Neutrophil (10^9/L)	0.689	0.637 - 0.741	6.845	48.8	81.8
Lymphocyte (10^9/L)	0.660	0.607 - 0.712	0.805	68.5	58.4
Platelet (10^9/L)	0.633	0.578 - 0.688	177.50	55.9	66.8
Albumin (g/L)	0.638	0.584 - 0.692	31.95	57.9	63.2
Urea (mmol/L)	0.654	0.601 - 0.708	4.95	79.9	55.0
CRP (mg/L)	0.675	0.623 - 0.728	70.0	62.0	66.4
SF (μg/L)	0.610	0.529 - 0.692	969.45	64.9	61.2
NT-proBNP (pg/mL)	0.692	0.634 - 0.751	503.5	53.8	78.1
IL-2R (U/mL)	0.613	0.525 - 0.701	1089.0	51.7	62.6
IL-6 (pg/mL)	0.663	0.582 - 0.743	41.42	61.0	68.7
IL-8 (pg/mL)	0.605	0.528 - 0.682	20.55	61.7	61.8
IL-10 (pg/mL)	0.625	0.553 - 0.697	5.05	83.1	44.5

SpO_2, pulse oxygen saturation; RR, Respiratory rate; WBC, White blood cell; CRP, C-reactive protein; SF, Serum ferritin; NT-proBNP, N terminal pro B type natriuretic peptide; CI, Confidence interval.

Supplementary Table 6. Single variable logistic regression model for predicting the risk of critical outcomes among male and female seriously ill COVID-19 patients. (only variables with p<0.1 were listed)

	Male			Female		
**Variables**	**Odds ratio**	**95% CI**	**p value**	**Odds ratio**	**95% CI**	**p value**
Age, per year	1.031	1.012 - 1.051	***0.001***	1.039	1.013 - 1.065	***0.003***
Diarrhea (yes vs no)	1.130	0.601 - 2.125	*0.703*	0.447	0.202 - 0.985	***0.046***
Fatigue (yes vs no)	1.272	0.810 - 1.997	*0.296*	0.557	0.313 - 0.994	***0.047***
Chest distress (yes vs no)	1.638	1.038 - 2.585	***0.034***	1.241	0.714 - 2.157	*0.445*
SPO_2 (< 95 vs ≥ 95)	1.143	0.694 - 1.881	*0.599*	2.297	1.224 - 4.310	***0.010***
RR, breaths per min (> 24 vs ≤ 24)	3.776	2.216 - 6.432	***<0.0001***	4.479	2.256 - 8.893	***<0.0001***
Hypertension (yes vs no)	1.639	1.048 - 2.562	***0.030***	0.861	0.498 - 1.488	*0.592*
Diabetes (yes vs no)	1.605	0.942 - 2.736	*0.082*	1.039	0.527 - 2.049	*0.912*
CKD (yes vs no)	8.114	0.835 - 78.873	*0.071*	1.161	0.235 - 5.721	*0.855*
Other disease (yes vs no)	1.343	0.777 - 2.320	*0.291*	1.802	0.961 - 3.376	*0.066*
WBC count, × 10^9/L						
4 - 10 vs ≤ 4	2.015	0.909 - 4.469	*0.085*	1.072	0.484 - 2.375	*0.864*
> 10 vs ≤ 4	5.496	2.224 - 13.580	***<0.0001***	4.156	1.694 - 10.192	***0.002***
Neutrophil count, × 10^9/L						
1.8 - 6.3 vs < 1.8	1.717	0.376 - 7.831	*0.485*	1.816	0.407 - 8.100	*0.434*
> 6.3 vs < 1.8	5.417	1.167 - 25.148	***0.031***	7.377	1.633 - 33.319	***0.009***
Lymphocyte count, × 10^9/L (≤ 0.8 vs > 0.8)	1.935	1.233 - 3.037	***0.004***	4.571	2.518 - 8.298	***<0.0001***
Platelet count, × 10^9/L (≤ 100 vs > 100)	5.708	2.459 - 13.249	***<0.0001***	3.780	1.556 - 9.183	***0.003***
Creatine kinase, U/L (> 185 vs ≤ 185)	2.373	1.161 - 4.851	***0.018***	1.436	0.455 - 4.531	*0.537*
LDH, U/L (> 220 vs ≤ 220)	3.540	1.362 - 9.207	***0.010***	2.352	0.893 - 6.196	***0.083***
$NaHCO_3$, mmol/L						

22-27 vs < 22	0.698	0.432 - 1.127	*0.141*	0.289	0.158 - 0.528	***<0.0001***
>27 vs < 22	1.064	0.439 - 2.581	*0.891*	0.634	0.275 - 1.466	*0.287*
Urea, mmol/L (> 7.5 vs ≤ 7.5)	3.043	1.842 - 5.029	***<0.0001***	2.406	1.262 - 4.589	***0.008***
CRP, mg/L						
10 - 50 vs < 10	1.346	0.585 - 3.095	*0.484*	3.281	1.051 - 10.243	***0.041***
> 50 vs < 10	2.483	1.191 - 5.176	***0.015***	7.821	2.684 - 22.789	***<0.0001***
PCT, ng/mL						
0.5 - 2 vs < 0.5	1.384	0.591 - 3.239	*0.454*	5.460	1.588 - 18.777	***0.007***
> 2 vs < 0.5	2.613	0.516 - 13.236	*0.246*	7.583	1.741 - 33.038	***0.007***
Hs-cTnI, pg/mL (> 28 vs ≤ 28)	4.415	2.419 - 8.059	***<0.0001***	6.050	2.965 - 12.344	***<0.0001***
NT-proBNP, pg/mL						
500 - 1000 vs < 500	2.348	1.148 - 4.804	***0.019***	3.392	1.505 - 7.645	***0.003***
> 1000 vs < 500	4.973	2.455 - 10.073	***<0.0001***	4.795	2.082 - 11.045	***<0.0001***
Fibrinogen, g/L						
2 - 4 vs < 2	0.697	0.158 - 3.076	*0.634*	0.128	0.020 - 0.802	*0.028*
> 4 vs < 2	0.332	0.081 - 1.364	*0.126*	0.130	0.023 - 0.735	***0.021***
D-Dimer, μg/mL						
0.5 - 1 vs < 0.5	1.084	0.477 - 2.465	*0.847*	2.243	0.744 - 6.764	*0.152*
> 1 vs < 0.5	2.815	1.423 - 5.572	***0.003***	4.275	1.610 - 11.348	***0.004***
IL-8, pg/mL (> 62 vs ≤ 62)	2.987	1.022 - 8.728	***0.045***	0.719	0.147 - 3.509	*0.683*
IL-10, pg/mL (> 9 vs ≤ 9)	1.476	0.740 - 2.943	*0.269*	2.657	1.155 - 6.116	***0.022***

SpO_2, Pulse oxygen saturation; RR, Respiratory rate; CKD, Chronic kidney disease; WBC, White blood cell; SF, Serum ferritin; Hs-cTnI, Hypersensitive cardiac troponin I; NT-proBNP, N terminal pro B type natriuretic peptide; PCT, Procalcitonin; CRP, C-reactive protein; LDH, Lactate dehydrogenase; CI, Confidence interval.

Supplementary Table 7. Single variable logistic regression model for predicting the risk of fatal outcomes among male and female critically ill COVID-19 patients (only variables with p<0.1 were listed)

	**Male**			**Female**		
**Variables**	**Odds ratio**	**95% CI**	**p value**	**Odds ratio**	**95% CI**	**p value**
Age, per year	1.035	0.998 - 1.074	*0.063*	0.994	0.947 - 1.044	*0.820*
Cough (yes vs no)	0.367	0.115 - 1.169	*0.090*	0.271	0.031 - 2.331	*0.234*
Hypertension (yes vs no)	0.557	0.232 - 1.338	*0.190*	0.375	0.126 - 1.119	*0.079*
WBC count, × 10^9/L						
4 - 10 vs ≤ 4	0.421	0.048 - 3.656	*0.433*	3.833	0.812 - 18.092	*0.090*
> 10 vs ≤ 4	0.317	0.034 - 2.976	*0.315*	42.000	3.667 - 481.030	***0.003***
Lymphocyte count, × 10^9/L (≤ 0.8 vs > 0.8)	1.444	0.605 - 3.447	*0.407*	2.691	0.854 - 8.479	*0.091*
Hs-cTnI, pg/mL (> 28 vs ≤ 28)	1.713	0.599 - 4.898	*0.315*	4.561	1.121 - 18.565	***0.034***
NT-proBNP, pg/mL						
500 - 1000 vs < 500	1.444	0.346 - 6.029	*0.614*	8.000	0.894 - 71.575	*0.063*
> 1000 vs < 500	1.667	0.467 - 5.947	*0.431*	2.444	0.542 - 11.028	*0.245*
D-Dimer, μg/mL						
0.5 - 1 vs < 0.5	0.733	0.137 - 3.938	*0.718*	20.000	1.391 - 287.600	***0.028***
> 1 vs < 0.5	1.167	0.282 - 4.826	*0.831*	11.000	1.115 - 108.488	***0.040***

WBC, White blood cell; Hs-cTnI, Hypersensitive cardiac troponin I; NT-proBNP, N terminal pro B type natriuretic peptide; CI, Confidence interval.

Supplementary Figure. 1

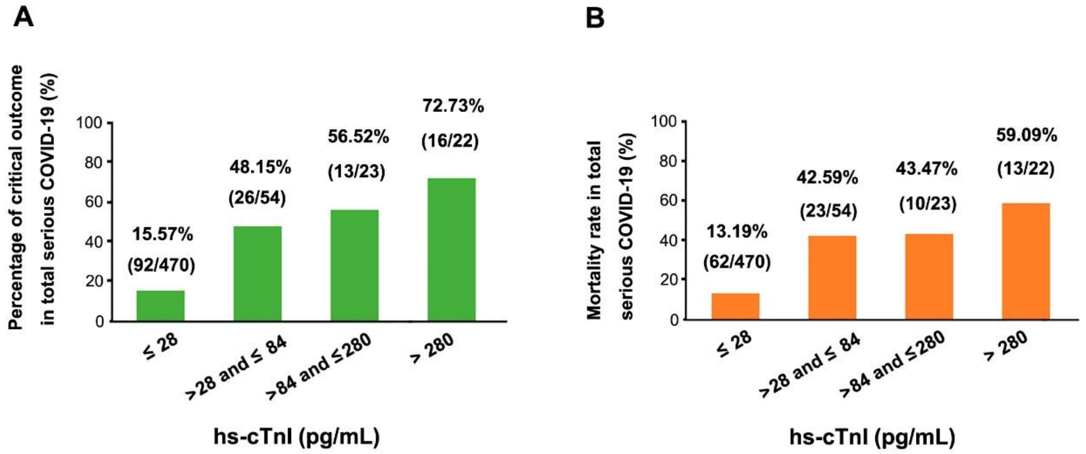

Supplementary Figure. 1. Percentage of critical outcomes (A) and fatal outcomes (B) among the total cases of serious COVID-19 at different concentrations of hs-cTnI. Approximately 78.37% (569/726) of patients with serious COVID-19 had serum hs-cTnI detected. A higher percentage of critical outcomes and fatal outcomes were accompanied by higher hs-cTnI concentrations. For serum hs-cTnI 28 pg/ml is 99th percentile upper reference limit (99th % URL). 84 pg/ml indicates 3 × 99th % URL of serum hs-cTnI; and 280 pg/ml indicates 10 × 99th % URL of serum hs-cTnI; COVID-19, Coronavirus disease 2019; hs-cTnI, Hypersensitive cardiac troponin I.

Supplementary Figure. 2

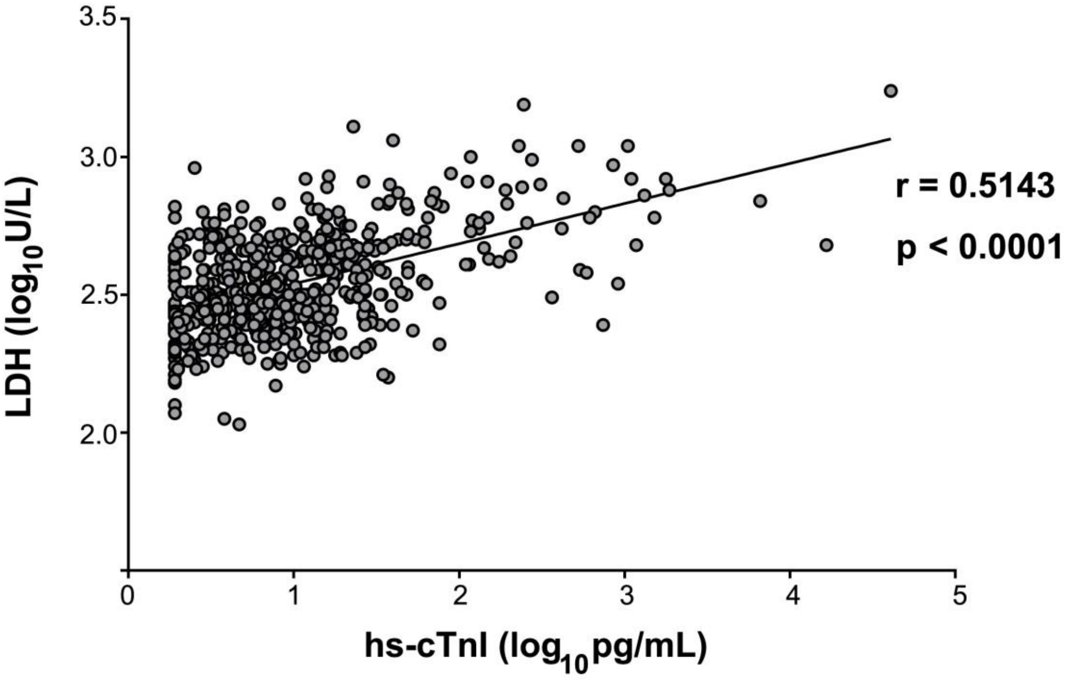

Supplementary Figure. 2. Correlation analysis between serum hs-cTnI and LDH concentrations among patients with serious COVID-19 in our study. In total, 568 serious cases had both hs-TnI and LDH detected. COVID-19, Coronavirus disease 2019; hs-cTnI, Hypersensitive cardiac troponin I. LDH, Lactate dehydrogenase.
